# Stabilization of c-KIT G-Quadruplex DNA Structures by the RNA Polymerase I Inhibitors BMH-21 and BA-41

**DOI:** 10.3390/ijms20194927

**Published:** 2019-10-04

**Authors:** Stefania Mazzini, Raimundo Gargallo, Loana Musso, Francesca De Santis, Anna Aviñó, Leonardo Scaglioni, Ramon Eritja, Massimo Di Nicola, Franco Zunino, Annabella Amatulli, Sabrina Dallavalle

**Affiliations:** 1 Department of Food, Environmental and Nutritional Sciences (DEFENS), Division of Chemistry and Molecular Biology, University of Milan, via Celoria 2, 20133 Milan, Italy; loana.musso@unimi.it (L.M.); leonardo.scaglioni@unimi.it (L.S.); Annabella.amatulli@gmail.com (A.A.); sabrina.dallavalle@unimi.it (S.D.); 2 Department of Chemical Engineering and Analytical Chemistry, University of Barcelona, Martí I Franqués 1-11, 08028 Barcelona, Spain; raimon_gargallo@ub.edu; 3 Unit of Immunotherapy and Anticancer innovative Therapeutics, Department of Medical Oncology Fondazione IRCCS, Istituto Nazionale dei Tumori, via Venezian 1, 20133 Milano, Italy; francesca.desantis@istitutotumori.mi.it (F.D.S.); massimo.dinicola@istitutotumori.mi.it (M.D.N.); francofzunino@gmail.com (F.Z.); 4 Institute for Advanced Chemistry of Catalonia (IQAC), CSIC, Networking Center on Bioengineering, Biomaterials and Nanomedicine (CIBER-BBN), Jordi Girona 18-26, E-08034 Barcelona, Spain; aaagma@cid.csic.es (A.A.); recgma@cid.csic.es (R.E.)

**Keywords:** c-KIT, G-quadruplex, pyridoquinazolinone derivatives, CD, NMR spectroscopy

## Abstract

The stabilization of G-quadruplex DNA structures by small molecules with affinity to oncogene promoters has emerged as a promising anticancer strategy, due to a potential role in gene expression regulation. We explored the ability of BMH-21 (**1**) and its analogue BA-41 (**2**) to bind the G-quadruplex structure present in the c-KIT promoter by biophysical methods and molecular modeling. We provide evidence that both compounds interact with the c-KIT 21-mer sequence. The stable monomeric intramolecular parallel G-quadruplex obtained by the mutation of positions 12 and 21 allowed the precise determination of the binding mode by NMR and molecular dynamics studies. Both compounds form a complex characterized by one ligand molecule positioned over the tetrad at the 3′-end, stabilized by an extensive network of π–π interactions. The binding constants (Kb) obtained with fluorescence are similar for both complexes (around 10^6^ M^−1^). Compound BA-41 (**2**) showed significant antiproliferative activity against a human lymphoma cell line, SU-DHL4, known to express substantial levels of c-KIT. However, the partial inhibition of c-KIT expression by Western blot analysis suggested that the interaction of compound **2** with the c-KIT promoter is not the primary event and that multiple effects provide a contribution as determinants of biological activity.

## 1. Introduction

Nucleic acids can fold into a variety of secondary structures [1]. Among them, those formed by guanine-rich sequences in the presence of monovalent metal ions are called G-quadruplexes [2]. The core of these structures consists of stacked planar arrangements called G-quartets formed by the association of four guanine bases through interactions of Hoogsteen and Watson−Crick faces of the adjacent guanines. G-quadruplex forming sequences have been identified in the promoter regions of many proto-oncogenes, such as c-*MYC*, c-*KIT*, *BCL-2*, k-*RAS*, *VEGF*, *HIF1α*, and *PDGF-A*, as well as at the end of telomeres, suggesting their relevant role in transcription regulation [3,4,5,6,7,8,9]. Thus, stabilization by small molecules of the G-quadruplex structures present in the promoter regions is a promising strategy to effectively inhibit the transcription process, and a new direction in anticancer drug discovery [10,11,12,13,14]. The c-*KIT* proto-oncogene encodes a transmembrane tyrosine kinase receptor (c-*KIT*) [14,15], which participates, after activation by endogenous ligands, in a broad range of biological events, including cell growth, proliferation, migration and survival [16]. The excessive activation of c- *KIT* or specific mutations in c-*KIT* have been implicated in a number of human cancers, such as gastrointestinal stromal tumors (GISTs), pancreatic cancer, melanoma and haematological neoplastic diseases [17,18].

Previous studies have shown that small molecules can effectively inhibit c-*KIT* kinase activity in vitro and in vivo [19,20]. However, resistance is emerging as a major clinical problem because of secondary mutations, amplification of c-*KIT*, and redundancy of signal/growth factors [15]. This supports the potential efficacy of multi-target therapy.

In the proximal promoter of c-*KIT*, three guanine (G)-rich regions have been identified that are able to fold into G-quadruplexes [5,16,21]. Stabilization of these structures by ligands in the promoter region of the c-*KIT* gene has been associated with inhibition of its transcriptional activity and reduction of the expression of c-*KIT* tyrosine kinase receptor, possibly leading to exploitable anticancer effects [22,23,24,25,26,27,28].

Therefore, the development of small molecules as c-*KIT* promoter G-quadruplex binding ligands can be considered as an alternative strategy to overcome the c-*KIT* protein mutation-related resistance.

**Scheme 1:**
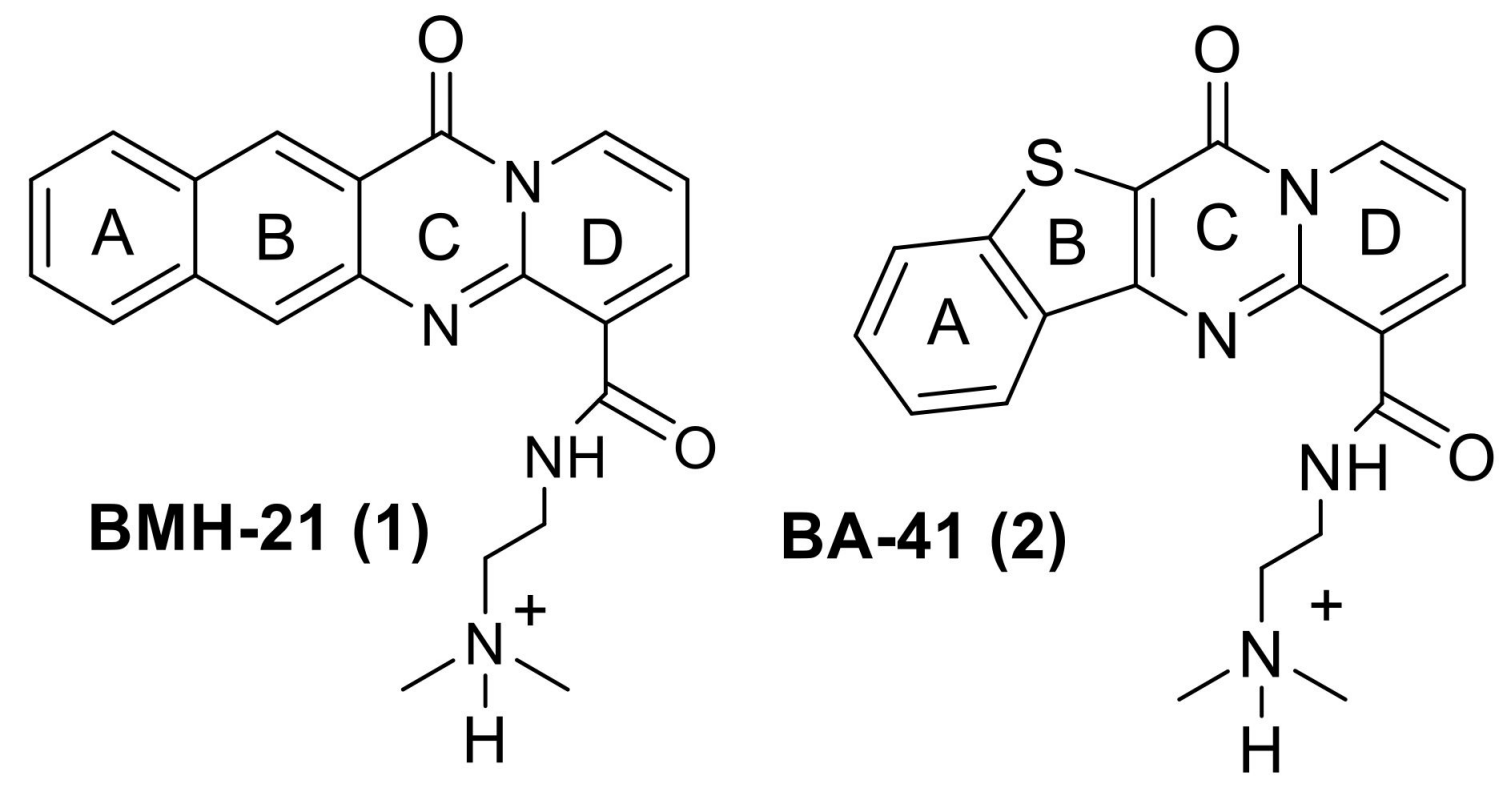
Chemical structures of G-quadruplex binders BMH-21 (**1**) and BA-41 (**2**).

BMH-21 (**1**) (Scheme 1) is a RNA polymerase I inhibitor that was described as a selective binder of GC-rich sequences of DNA [29]. We have recently explored the ability of BMH-21 (**1**) and its analogue BA-41 (**2**) (Scheme 1) to bind to G-quadruplexes. We have provided evidence that both compounds are not DNA intercalators but are effective binders of the human telomeric and c-MYC promoter G-quadruplexes [30].

Based on these observations, the main purpose of the present study was to extend previous findings and to investigate the ability of compounds **1** and **2** to interact with the c-KIT G-rich promoter sequence. Biophysical methods including NMR, circular dichroism (CD) spectroscopy, molecular absorption (UV) spectroscopy, and fluorescence titration experiments were used to evaluate the interactions of the compounds with the G-quadruplex structures of the c-*KIT* promoter. Molecular modeling was also employed to investigate the binding mode of **1** and **2** with this sequence. Furthermore, the biological effects of compounds were investigated in cell models expressing substantial levels of c-*KIT*.

## 2. Results and Discussion

The c-kit2 21-mer sequence, 5′-CGGGCGGGCGCGAGGGAGGGG-3′, adopts in K^+^ solution two distinct parallel G-quadruplex conformations in slow exchange, which can form either a monomeric or a dimeric structure, depending on the concentration of KCl [31,32]. It was found that the G to T mutation at positions 12 and 21 (c-kit21T12T21: 5′-CGGGCGGGCGCTAGGGAGGT-3′) further stabilizes, at low KCl concentration, the monomeric intramolecular parallel G-quadruplex [31,32]. Several authors have used the c-kit21T12T21 oligonucleotide to study the binding properties of G-quadruplex binding drugs by NMR, as the wild sequence gives poorly resolved proton NMR data [33,34]. It has been demonstrated that the thermal stabilization of six ligands is similar in both wild type and mutated (c-kit21T12T21) sequences [33].

The purine-rich nuclease hypersensitivity element III1 (NHE III1) of c-MYC, which controls 80–90% of c-MYC transcription, is a 27-nt segment (MYCPu27), containing five consecutive runs of guanines [4,35]. Pu22 is a 22-mer sequence of MYCPu27, mainly responsible for c-MYC transcriptional activity. Recently, it has been reported that Pu22-T14T23, with two G-to-T mutations at positions 14 and 23, gives the same interaction with ligands as wild-type Pu22 [36]. Thus, these mutated sequences, namely c-kit21T12T21 and Pu22T14T23, are suitable for spectroscopic experiments.

### 2.1. CD, UV and Fluorescence Results

The spectroscopic studies were performed with the mutated sequence c-kit21T12T21 in comparison with the Pu22T14T23 sequence of the c-MYC promoter.

**Figure 1:**
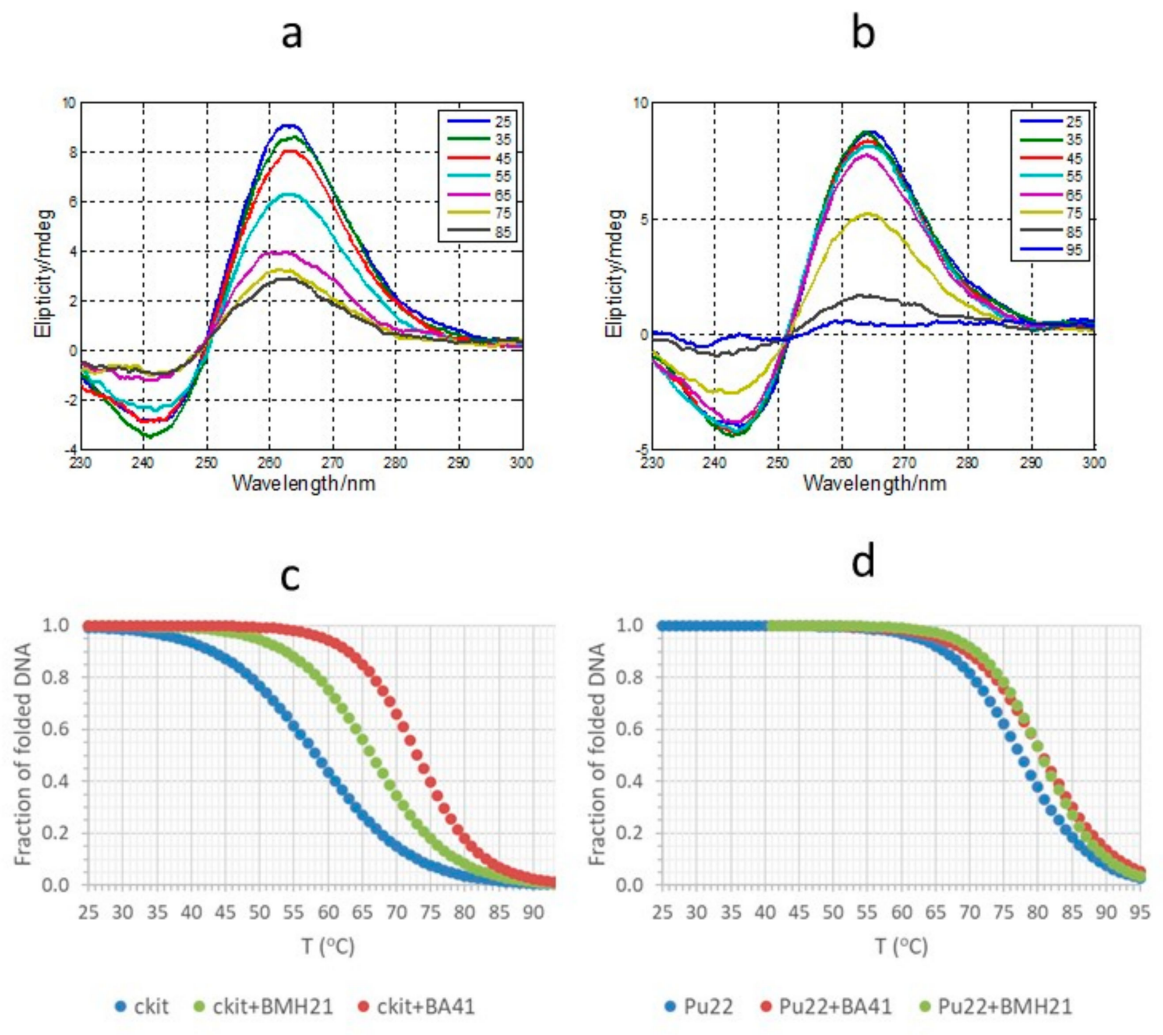
Circular dichroism (CD) spectra recorded along the thermally induced unfolding of c-kit21T12T21 (**a**) and Pu22T14T23 (**b**). Fraction of folded DNA vs. temperature in the absence and presence of the ligands calculated from the CD trace at 262 nm: (**c**) BMH-21 (**1**) and BA-41 (**2**) with c-kit21T12T21, and (**d**) BMH-21 (**1**) and BA-41 (**2**) with Pu22T14T23. The medium consisted of 5 mM phosphate buffer (pH 7.0) and 20 mM KCl. DNA and ligand concentrations were 1 μM and 3 μM, respectively.

The CD spectra of c-kit21T12T21 and Pu22T14T23 sequences alone showed a negative band at 245 nm and a positive band at 262 nm. These bands are characteristic of a parallel G-quadruplex topology (Figure 1a,b). Upon heating, these structures unfolded. The shape and intensity of the CD spectrum of Pu22T14T23 was practically lost at 85 °C. However, this did not occur for c-kit21T12T21, which could be indicative of the presence of multimeric structures, other than the monomeric G-quadruplex one [31,32]. This hypothesis is based on a previous work with a similar sequence showing that the residual CD signal was related to the presence of multimeric forms that were stable at this temperature [37]. At this point, size-exclusion chromatography (SEC) measurements provided a complementary insight into the polymorphism of these two sequences [37,38,39].

**Figure 2:**
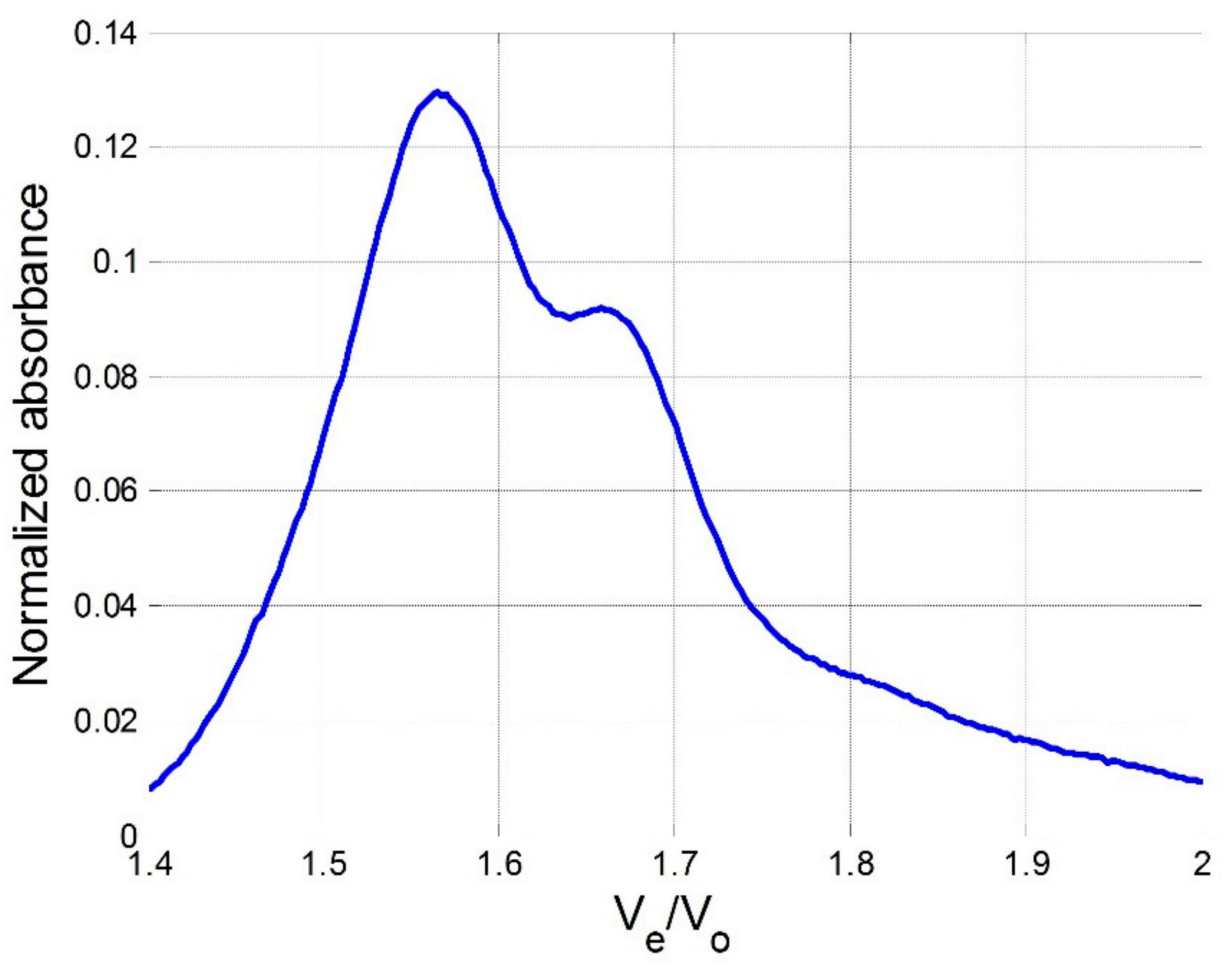
Size-exclusion chromatogram of c-kit21T12T21. The assignation of bands has been done according to the ratio elution volume/void volume, calculated according to reference [38].

The chromatogram for c-kit21T12T21 (Figure 2) showed the presence of two well-resolved bands at elution volume/void volume (V_e_/V_0_) ratios equal to 1.57 and 1.66, respectively. According to the calibration plot built by Largy and Mergny [38], these two bands correspond to a dimer and to a monomer structure. The presence of these two structures has been already described by other authors [31,32] based on NMR studies. Finally, the chromatogram for Pu22T14T23 showed two clearly resolved bands at V_e_/V_0_ ratios equal to 1.62 and 1.75, respectively (Figure S1). According to the referred calibration plot, these two bands were assigned to a monomer and an unfolded sequence.

**Figure 3:**
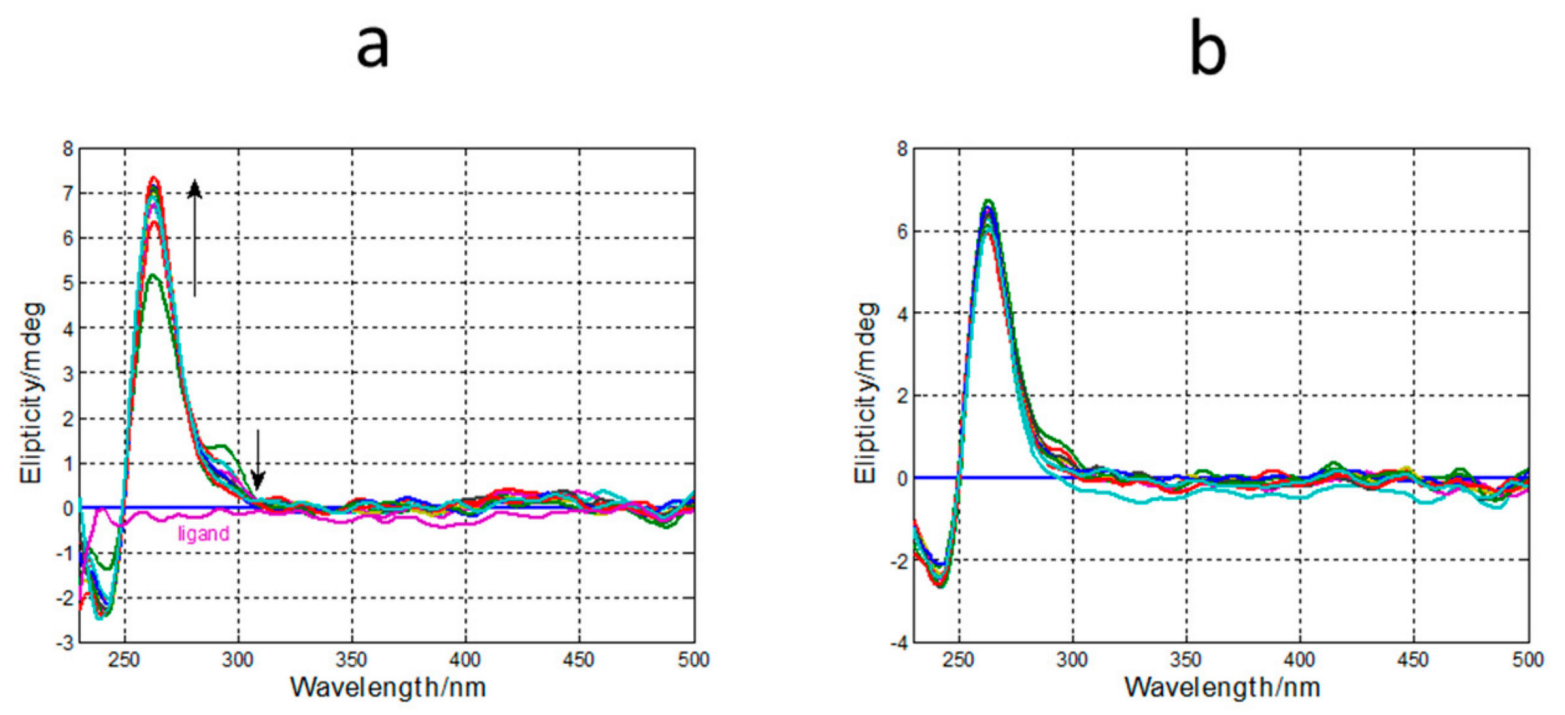
CD spectra recorded along the titration of c-kit21T12T21 with (**a**) BMH-21 (**1**) and (**b**) BA-41 (**2**). In both cases, the medium consisted of 20 mM phosphate buffer (pH 7.0) and 5 mM KCl. DNA concentration was 1 μM. Ligand concentration ranged from 0 to 3 μM. Experiments were carried out at 25 °C.

The addition of ligands did not affect the shape and intensity of the CD spectra of both G-quadruplex sequences recorded in the UV region (Figure 3 and Figure S2). This result proves the preservation of the G-quadruplex structure upon interaction with the ligand; moreover, no induced CD (ICD) signals appeared in the visible region after treatment with both compounds **1** and **2**. As ICD signals are usually related to intercalation [40], the absence of ICD suggests a mode of binding weaker than intercalation, such as end-stacking or electrostatic interaction.

The thermodynamic parameters calculated, assuming a two-state process, from the ellipticity changes at 262 nm (see Figure 1c,d) are summarized in Table 1. The folded structure formed by Pu22T14T23 complexes is more stable than that formed by c-kit21T12T21 complexes, not only in terms of T_m_ values but also in terms of changes in Gibbs free energy at 37 °C. This result could be explained by the formation in the Pu22T14T23 sequence of a parallel propeller-like G-quadruplex with short double-chain reverse loops. Shorter loops are related to higher thermal stability of G-quadruplex structures [41,42,43]. In this case, c-kit21T12T21 has a long –CGCTA-loop in the middle of the sequence compared with the shorter –TA-loop found in a similar position on the Pu22T14T23 sequence. This could be one of the reasons for the lower stability of c-kit21T12T21.

In spite of the lower thermal stability of the c-kit21T12T21 quadruplex, the binding of ligands **1** and **2** induced a large increase in T_m_ values and Gibbs free energy at 37 °C. Especially relevant is the stabilization observed upon the binding of ligand **2**, with an increase in T_m_ of 14.9 °C compared with the increase in T_m_ of 8.2 °C for ligand **1**. A possible explanation for these differences is the interaction of the quaternary nitrogen atom of ligand **2** with the NH group at the C5 unit, which is not found in ligand **1**, as explained in detail below (Section 2.5 Molecular Modeling).

**Figure 4:**
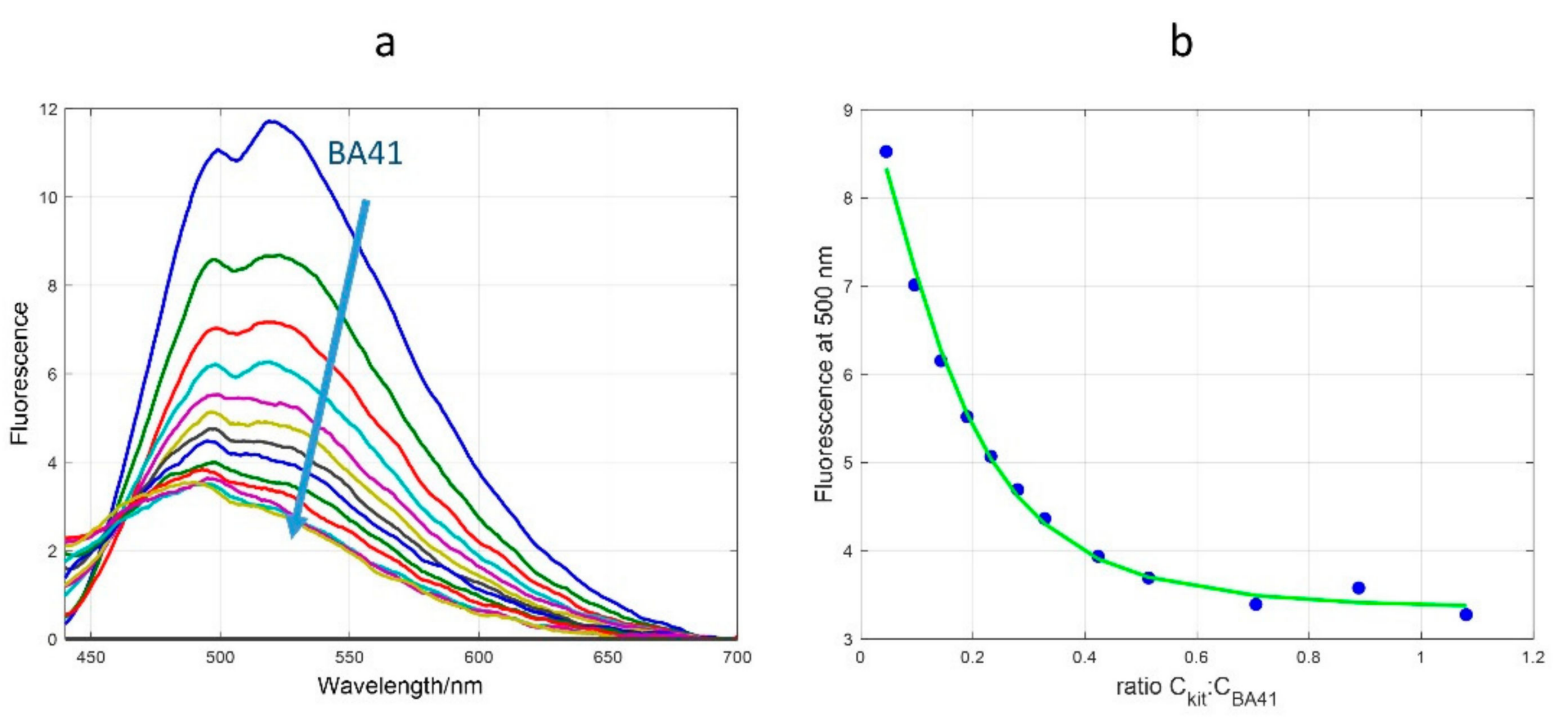
(**a**) Fluorescence spectra recorded along the titration of BA-41 (**2**) with c-kit21T12T21. Original fluorescence signal at 500 nm for the titration of BA-41 (**2**) at different concentrations of c-KIT (**b**).

**Figure 5:**
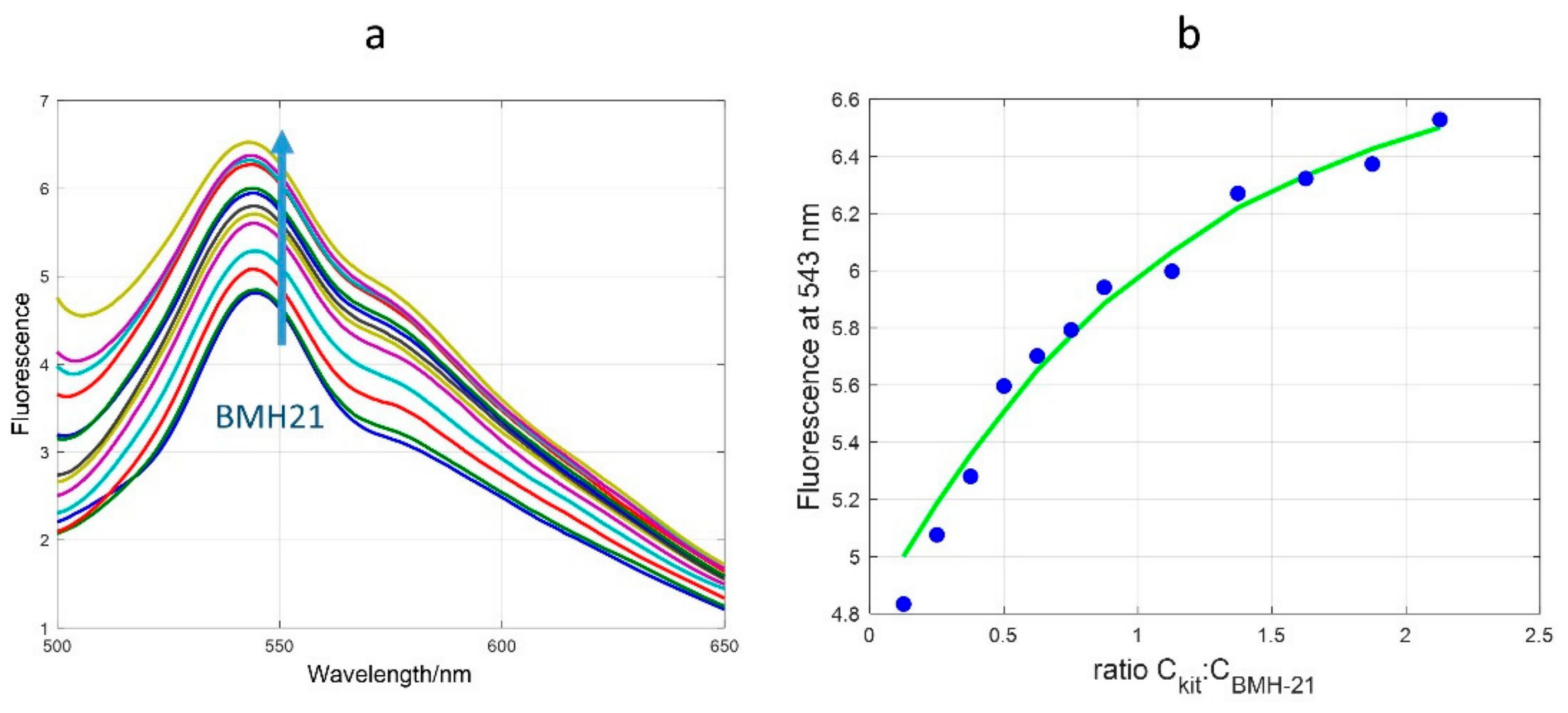
(**a**) Fluorescence spectra recorded along the titration of BMH-21 (**1**) with c-kit21T12T21. (**b**) Original fluorescence signal at 535 nm for the titration of BMH-21 (**1**) at different concentrations of c-kit21T12T21.

The fluorescence spectra of both ligands in presence of the c-kit21T12T21 sequence are reported in Figure 4; Figure 5. Both compounds showed fluorescence in buffer solution without oligonucleotides. However, the addition of the oligonucleotide induced different changes in the fluorescence signal intensity. In the case of BA-41, a decrease in the fluorescence intensity was observed upon addition of the oligonucleotide, whereas a small increase was observed in the case of BMH-21. From the titration curves, an estimation of the stoichiometry and the binding constants (Kb) relative to the interaction of these ligands with c-kit21T12T21 was carried out (Table S1). We used the EQUISPEC program based on the multivariate analysis of all the spectra measured along the titration [44]. In both cases, the Kb values obtained for **1** and **2** with c-kit21T12T21 were in the order of 10^6^ M^-1^, which suggests a relatively strong interaction between both ligands and this sequence, higher than the interaction with Pu22T14T23 (Kb *ca* 10^5^ M^−1^) [30].

### 2.2. Interaction of **1** and **2** with the c-kit21T12T21 Sequence by NMR 

c-kit21T12T21  5′- C G G G C G G G C G C **T^12^**A G G G A G G G **T^21^**-3′ 

Pu22T14T23   5′- T G A G G G T G G G **T^14^** A G G G T G G G **T^23^** A A -3′

**Figure 6:**
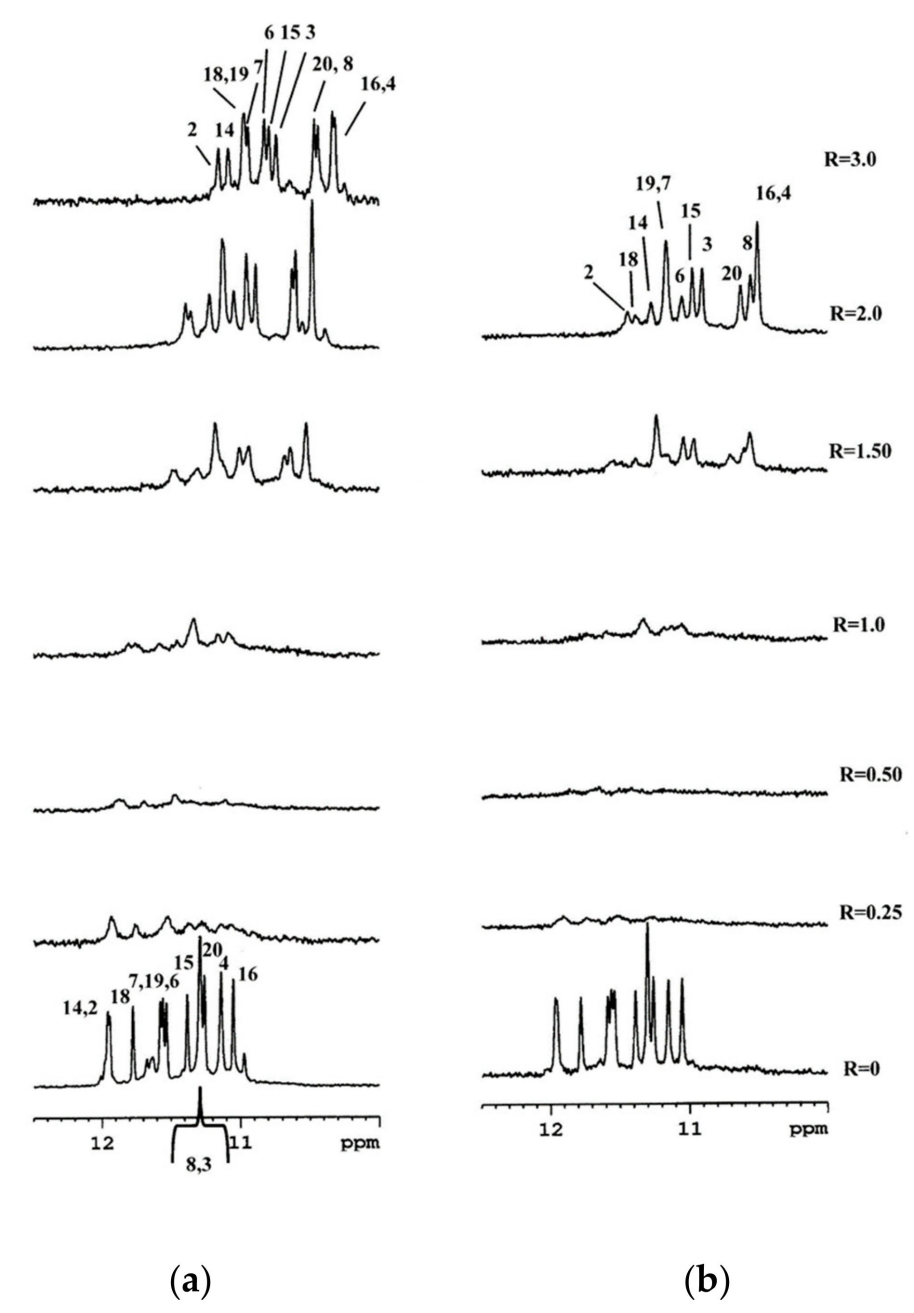
Imino proton region of the 1D NMR titration spectra of c-kit21T12T21 with (**a**) **2** and (**b**) **1** at 25 °C in H_2_O/D_2_0 (9:1), 5 mM KH_2_PO_4_, 20 mM KCl, pH 6.9, at different *R* = [drug]/[DNA] ratios.

**Figure 7:**
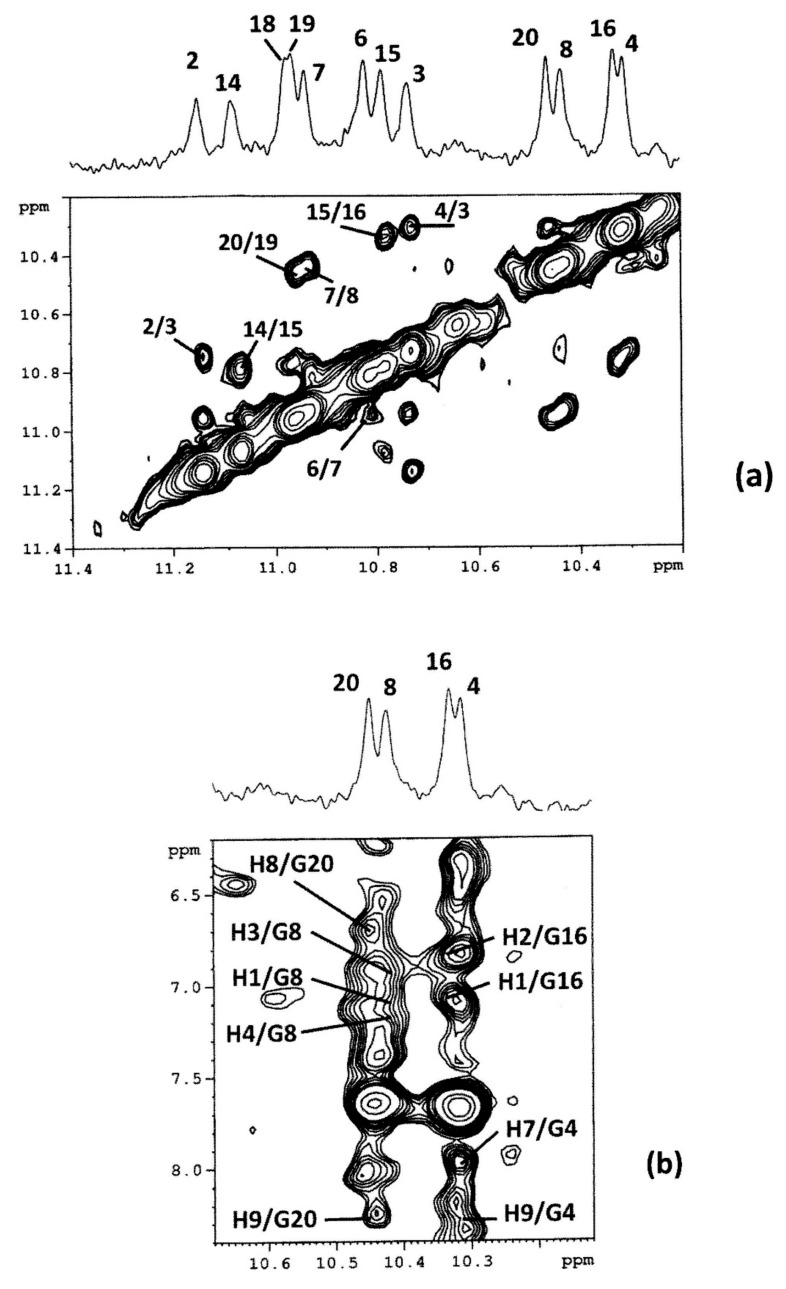
Selected region of the 2D NOESY spectrum of the c-kit212T12T21/(**2**) complex. (**a**) Sequential NOE interactions in the H1 region and (**b**) the intermolecular NOEs between H1 imino protons and (**2**) H aromatic protons.

**Scheme 2:**
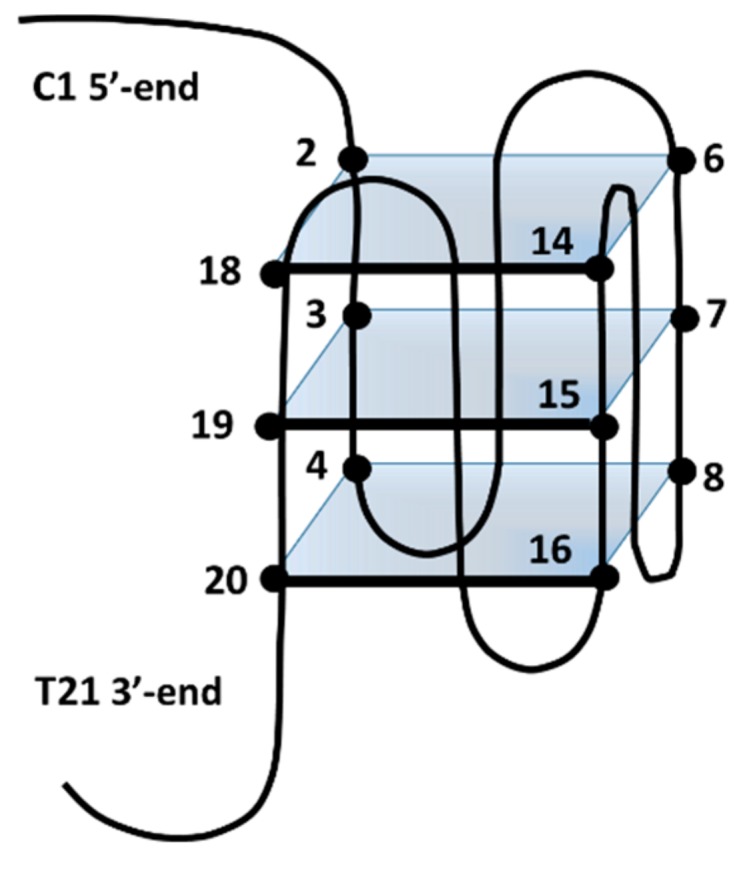
Scheme of the potential structure for the c-kit21T12T21 quadruplex.

The addition of both ligands to c-kit21T12T21 solution induced significant changes in the ^1^H *NMR* spectrum, even at low ratio *R* = [ligand]/[DNA] = 0.25/1.0 (Figure 6). The common trend was an upfield shift and a generalized broadening of H1 imino protons with increasing concentration of the ligands. The signals sharpened at *R* = 1.50–2.0, suggesting the formation of a well-defined complex. The H1 imino protons, remaining in a region ranging from 10.3 to 11.5 ppm, indicate that the G-quadruplex structure is conserved. The resonances of the complex with **1** were in general broader than those with **2**, and the proton signals of both ligands remained very broad during all the titration experiments. The assignment of the nucleotide sequence in the spectra of both complexes was performed following the known procedure, e.g., the cross-checking between imino and aromatic protons through their NOE contacts, with the help of the sequential NOE interactions in the H1 region (Figure 7a). This allowed the assignment of the guanine protons. The inter-residue NOE connectivities of these resonances, characteristic of the three tetrads, were all detected. For instance, G4H1/G8H8, G8H1/G16H8, G16H1/G20H8, and G20H1/G4H8 define the plane of tetrad I. The two other planes, tetrads II and III, were determined in the same way (see Table S2, Scheme 2).

The identification of the cytidine aromatic protons was based on TOCSY experiments, as the vicinal H6 and H5 protons displayed very strong signals. Their assignment was possible because the shift remained constant vs. the free nucleotide. The thymine protons were very easily identified through the methyl resonances, lying upfield. Then, the strong NOEs between methyl and H6 protons allowed us to distinguish the aromatic protons of the thymines from those of the adenines. The H8 protons of the latter lied downfield, as well as their anomeric H1’ protons, both resonances being constant vs. the free nucleotide. The H2 adenine protons, instead, were not identified. Finally, the assignment of T12 vs. T21 was possible through the presence of a sequential NOE of A13 H8 with T12 Me and T12 H2’ ribose protons.

The interaction of both ligands with c-kit21T12T21 induced a large change in the chemical shifts of H1 imino protons: in presence of BA-41 at *R* = 3.0, Δδ ≥ 0.80 ppm for the imino protons belonging to outer G-tetrads and Δδ ≤ 0.60 ppm for the internal tetrad. The same trend was observed for the complex with BMH-21 (Table S3, Table S4, Graph S1 and Graph S2).

The shift variation of the guanine imino protons of the external G tetrads is the first evidence of the location of the ligand. On the other hand, the upfield shift also observed for the imino protons belonging to the internal tetrad is difficult to explain, but this can suggest an external interaction at the level of this tetrad. None of the NOE cross peaks between the imino protons of the stacked guanine residues in the quadruplex structure disappeared after the addition of the ligands, clearly confirming that the ligands do not intercalate into the quadruplex.

### 2.3. BA-41 (**2**) Binds the c-kit21T12T21 Sequence,Where One Molecule is Positioned at the Tetrad at the 3-′end 

The intermolecular NOE interactions gave evidence of a stable position of the ligand. NOE contacts were found between the aromatic protons of ring A (H1-H4) and the imino protons of guanines G8 and G16, while other significant NOEs interactions involved the protons of ring D, i.e., H7, H8 and H9 with H1 G4 and H1 G20 (Table 2 and Figure 7b). These results indicate the stable position of **2** on the tetrad at the 3′-end, while the interaction at the 5′-end seems to be characterized by a location outside of tetrad III, or by a high mobility of the ligand, thus precluding NOE interactions. 

The loop chains did not show any contact with the ligand. Only a small shift variation was observed except for T21, C1 and C9 protons. The deshielding of T21 Me and T21 H6 is significant (∆δ +0.20 ppm and +0.36 ppm, respectively), indicating that this unit at the 3′-end is pushed away from the G tetrad I, thus no longer being stacked with the G20 unit. This is confirmed by the absence of a NOE between T21 Me and G20 H8, which is present in the free nucleotide. A similar deshielding was observed for H6 C9 (∆δ +0.23 ppm), showing a change in the orientation of this unit, which is no longer stacked with the aromatic portion of C5, as occurs in the free oligonucleotide.

This is in agreement with the model (see Section 2.5). Thus, the change in the conformation at the 3′-end is due to the location of the ligand at this site.

The deshielding of C1 H5 (Δδ +0.55 ppm) is also due to a change in conformation, as this unit is no longer stacked with the A13 aromatic ring, as in the free nucleotide. In the complex, A13 moves toward the G2 unit, as it results from the NOE between G2 H8 and A13 H8. These results showed that the ligand induces some perturbation in this site, but cannot enter into the tetrad because the site is protected by the loop.

### 2.4. Interaction of BMH21 (1) with c-kit21T12T2

The shift variation of the guanine H1 imino protons (Table S3) in the tetrads is comparable to those observed in the complex with **2**, thus suggesting a similar location of the ligand. However, the intermolecular NOE interactions were poor, in part due to the difficulty of detecting the resonances of the ligand because of their strong broadening. Some NOE contacts with the guanine of the tetrad at the 3′-end involve the protons of ring A (H1–H4), which remain unassigned. On the contrary, H10 and H9 of ring D, identified at 8.08 and 6.13 ppm, did not present any NOE interactions. Thus, the molecular modeling was built without specific NOE interactions.

### 2.5. Molecular Modeling

**Figure 8:**
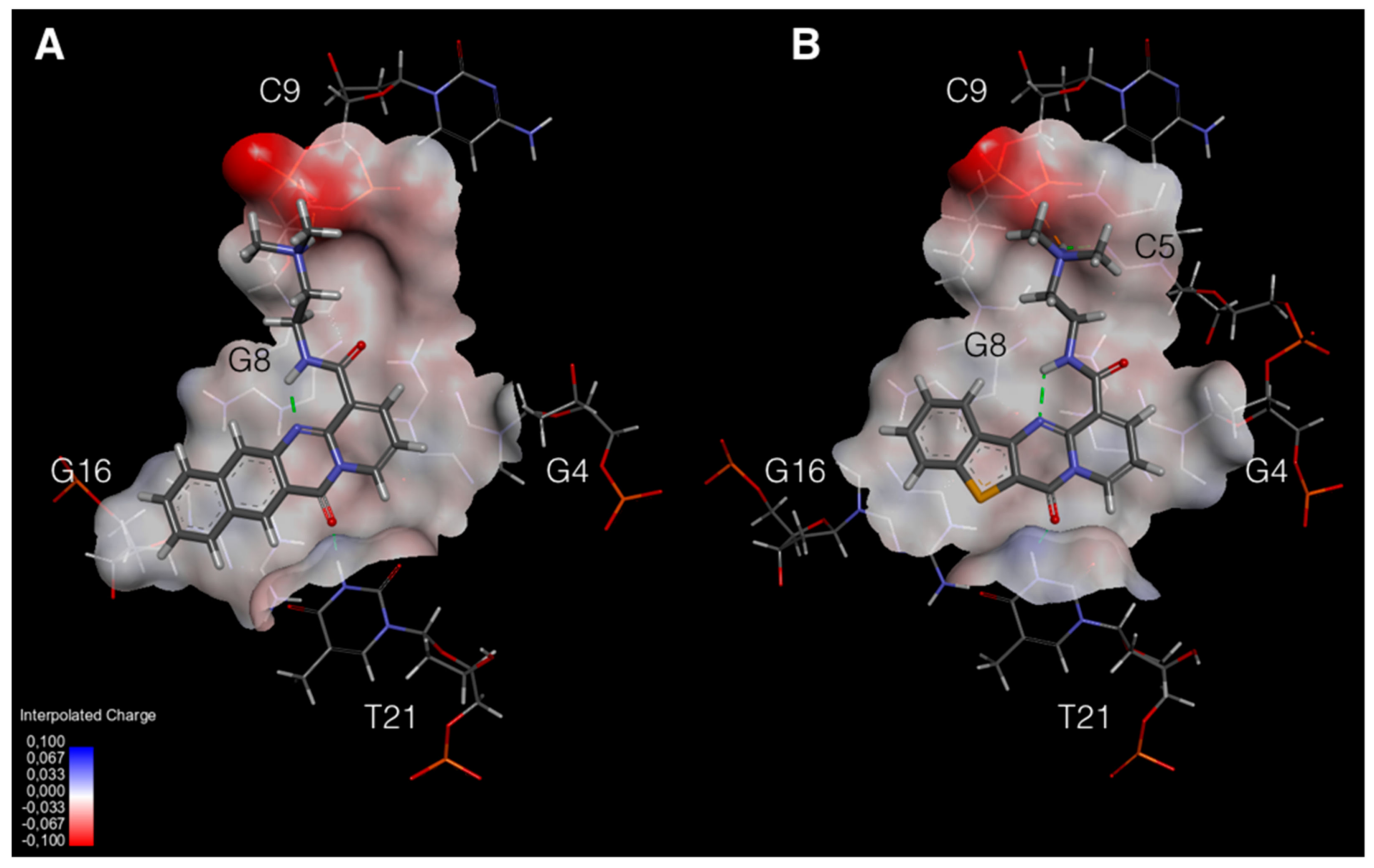
Molecular models of the optimized complexes of **1** (**A**) and **2** (**B**) with c-kit21T12T21 at the 3′-end G-tetrad binding sites. Ligands are rendered with sticks while bases are with lines, both colored by atoms. Solvent accessible surfaces (SAS) are coded following the lower left scale in the figure. Hydrogen bonds are represented by dashed green lines.

To visualize the binding mode of **1** and **2** to c-kit21T12T21, we performed a docking study followed by molecular mechanics calculations. Molecular modeling studies were conducted following the protocol described in the Materials and Methods section. The best docked conformations of **1** and **2** in the c-KIT complexes are very similar and in good agreement with the reported NOE contacts (Table 2). Both compounds are stabilized by an extensive network of π–π interactions involving the underlying 3′-end G-tetrad, with ring C located near the center of the tetrad. The differences observed in the two complexes are mainly due to the different topologies of the two polycyclic systems. In compound **2**, the aromatic ring A interacts with the π system of G8, while ring C moves away from G8 itself (Table S5). In compound **1** meanwhile, ring A is located over the G16 unit. The interactions involving the aromatic system are completed by a hydrogen bond between the carbonyl group of ring C and the NH group of T21, with distances of 1.57 Å and 1.67 Å for **1** and **2**, respectively (Figure 8).

The side chain in both compounds is arranged to allow the formation of a salt bridge between the quaternary nitrogen atom of the ligand and the phosphate group of the C9 unit. In the complex with **2**, the quaternary nitrogen atom of the ligand is also able to form a weak hydrogen bond with the NH group of the C5 unit, at a distance of 2.45Å. This interaction is not found in compound **1**. These different behaviors of the two ligands can be explained by the presence of an intramolecular hydrogen bond between the amide NH of the side chain and the nitrogen of ring C. This intramolecular interaction limits the conformational freedom of the side chain in the complex with **1**, and this causes the quaternary nitrogen to move away from the C5 unit.

### 2.6. Biological Activity

**Figure 9:**
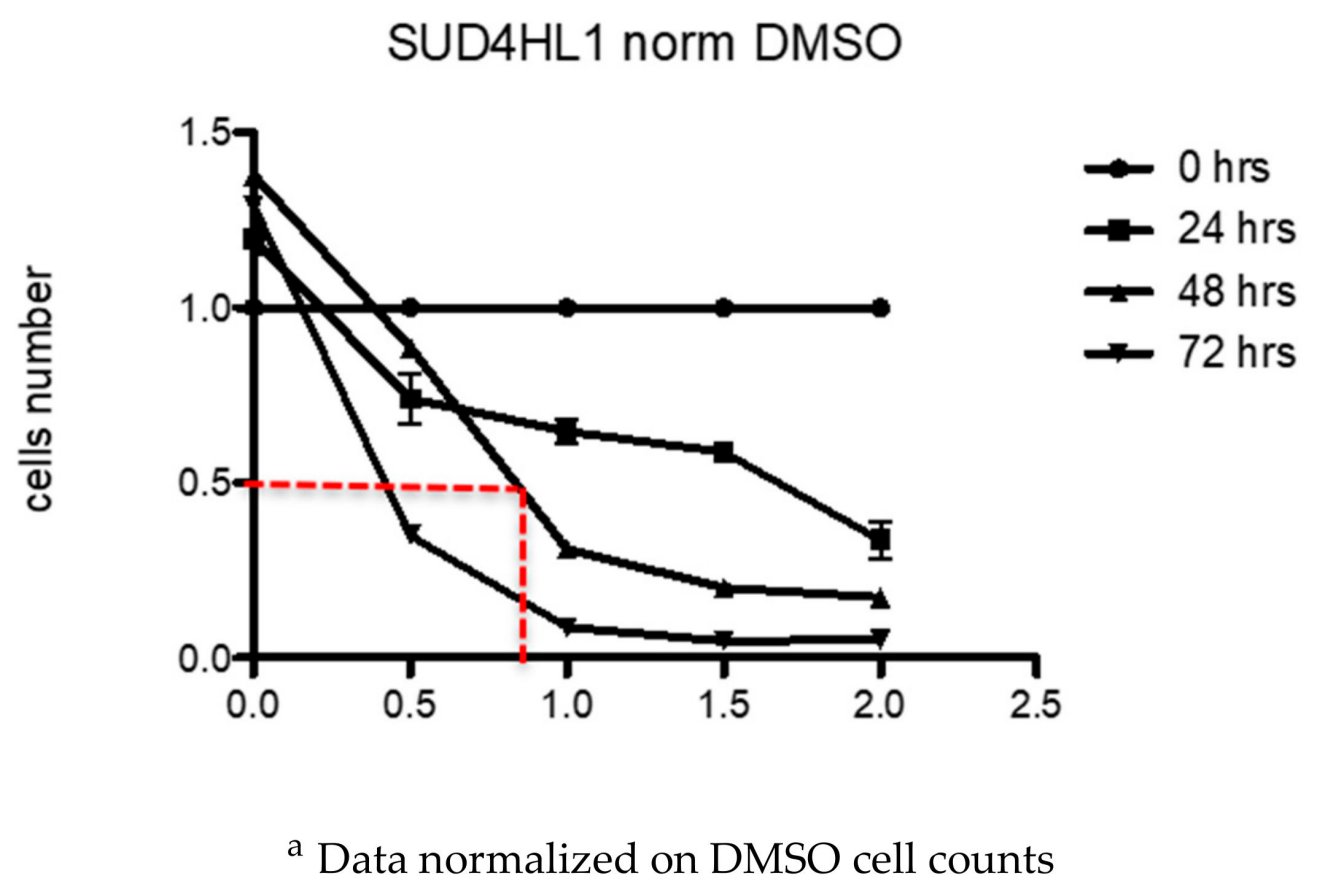
Determination of IC_50_ of compound **2** in SU-DHL4 cells (0.8 μM, 48 h; 0.45 μM, 72 h). Data are normalized on DMSO cell counts.

The biological/biochemical activity of the most promising compound, **2**, was studied in a human lymphoma model, known to express substantial levels of c-KIT. In particular, to calculate the concentration of the drug able to inhibit the proliferation of 50% of the cell population (IC_50_), treatment of the cell line SU-DHL4 was performed with compound **2** in dimethyl sulfoxide (DMSO, Mylan) at different concentrations, obtained with serial dilutions: 0.5, 1, 1.5 and 2 µM. As expected, the lymphoma cells were found to be responsive to the tested compound (IC_50_ ca 0.8 μM) (Figure 9). 

**Figure 10:**
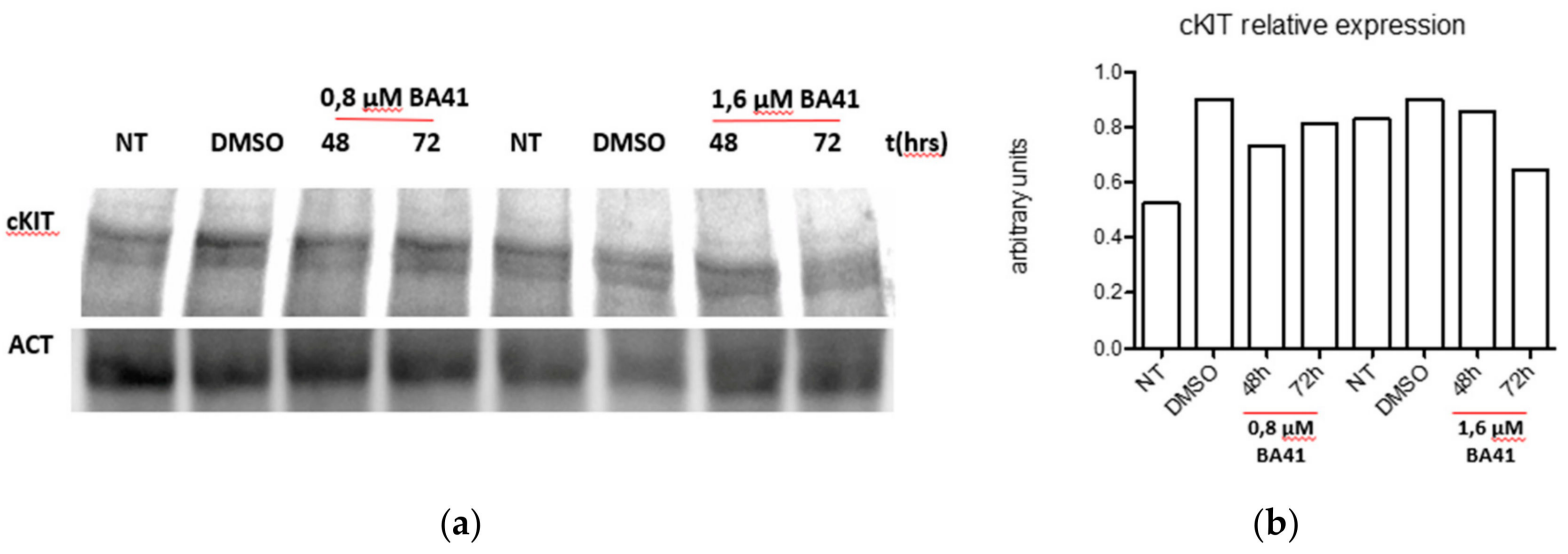
(**a**) Western blot analysis representing c-*KIT* expression in SU-DHL4 cells treated with BA-41 (**2**) (0.8 and 1.6 μM). β-actin was used as an endogenous normalizer of the amount of loaded proteins. (**b**) Densitometric analysis performed using ImageJ software (http://rsbweb.nih.gov/ij/).

In spite of the potent antiproliferative activity, the Western blot analysis documented only a partial inhibition of c-*KIT* expression (Figure 10a,b). This observation suggests that the interaction of the compound **2** with the c-*KIT* promoter is not the primary event and that multiple effects provide a contribution as determinants of biological activity. Indeed, the key role of c-*KIT* in controlling survival, proliferation and differentiation suggests the existence of a finely tuned network of molecular interactions, as documented by our present and previous results, which show the multitarget effects mediated by G-quadruplex stabilization [30]. Given the multitarget mechanism of action of these compounds and the different impacts of modulation of putative targets in tumor cells, no close correlations could be expected between the expression of each target and the effective concentration. Indeed, the chemosensitivity of the tested cell lines, expressing different targets, was found in a comparable range of concentrations [30].

## 3. Materials and Methods

### 3.1. Ligands

Compounds **1** and **2** were synthesized as previously described [30].

### 3.2. CD and Fluorescence Experiments

CD spectra were recorded on a Jasco J-810 spectropolarimeter equipped with a Peltier temperature control unit (JASCO, Seelbach, Germany). The DNA solution (c-kit21T12T21 or Pu22-T14T23) was transferred to a covered cell, and ellipticity was recorded with a heating rate of approximately 0.4 °C·min^−1^. Simultaneously, CD spectra were recorded every 5 °C from 210 to 320 nm. The spectrum of the buffer was subtracted. Each sample was allowed to equilibrate at the initial temperature for 30 min before the melting experiment began. In all experiments, the concentration of DNA was kept constant (1 µM), whereas the concentration of the considered ligands was increased. The medium consisted of 5 mM KH_2_PO_4_ and 20 mM KCl.

CD data recorded at 262 nm as a function of temperature were analyzed as described elsewhere [45]. For the unfolding of intramolecular structures such as those studied here, the chemical equation and the corresponding equilibrium constant may be written as:

DNA _folded_ + heat ↔ DNA _unfolded_ K_unfolding_ = [DNA _unfolded_]/[DNA _folded_] (1)

For melting experiments, the concentration of the folded and unfolded forms is temperature-dependent. The equilibrium constant depends on temperature according to the van’t Hoff equation:

ln K _unfolding_ = − ∆H_vH_/RT + ∆S_vH_/R (2)

It is assumed that ∆H_vH_ and ∆S_vH_ do not change throughout the range of temperatures studied here.

Molecular fluorescence spectra were measured with an AMINCO-Bowman AB2 spectrofluorimeter (Thermo Fisher Scientific, Waltham, MA, USA). The temperature was controlled by means of a water bath. Measurements were taken at 450 and 380 nm excitation wavelength for **1** and **2**, respectively. In all spectroscopic studies, Hellma quartz cells (10 mm path length and 350 µL volume) were used. The medium consisted of 5 mM phosphate buffer (pH 7.0) and 20 mM KCl. In all experiments, the concentration of ligands was kept constant (2 or 5 µM), whereas the concentration of the considered DNA sequence was increased.

The determination of the stoichiometries and the calculation of the binding constants was done from the fluorescence data recorded along titrations of ligands with DNAs by using the EQUISPEC program [44]. This program is based on the multivariate analysis of all the spectra measured along the titration. Briefly, it decomposes the data matrix containing these spectra into three matrices (C, S, and E), which contain the calculated concentration profiles, pure spectra and residuals for the proposed model of species. 

### 3.3. Size-Exclusion Chromatography (SEC)

The chromatographic system consisted of a Waters 2695 HPLC instrument (Waters Corp., Milford, USA). equipped with a quaternary pump, a degasser, an autosampler, a photodiode-array detector with a 13 μL flow cell, and software for data acquisition and analysis. The chromatographic column used for separation at room temperature was PSS Suprema Analytical Lineal S 100−100.000 Da (PSS Polymer Standards Service GmbH, Mainz, Germany). The composition of the mobile phase was 300 mM KCl and 20 mM phosphate (pH 7.1). The flow was set to 0.8 mL·min^−1^. The injection volume was 15 μL. T_15_, T_20_, T_25_, T_20_ and T_45_ sequences were used as standards to construct the plot of logarithm of the retention time (t_R_) vs. molecular weight [37]. Blue dextran (MW 2,000,000 Da, Sigma-Merck, Darmstadt, Germany) was used as a void volume marker (5.30 mL).

### 3.4. Nuclear Magnetic Resonance Experiments

c-kit21T12T21: 5′-CGGGCGGGCGC**T**AGGGAGGG**T-3′**

The sample of c-kit21T12T21 was prepared at a concentration of 0.42 mM, dissolved in 5mM KH_2_PO_4_, 20 mM KCl, pH 6.9. The DNA sample was heated to 85 °C for 1 min and then cooled at room temperature overnight. Stock solutions of **2** and **1** were prepared in DMSO-*d6*. ^1^H NMR titrations were performed at 25 °C by adding increasing amounts of ligand to the DNA at different ratios of R = [drug]/[DNA]. The protons in the complexes were assigned by using NOESY and TOCSY experiments. Phase-sensitive NOESY spectra were acquired at 25 °C, in TPPI mode, with 2048 × 1024 complex FIDs. Mixing times ranged from 100 to 300 ms. TOCSY spectra were acquired with the use of a MLEV-17 spin-lock pulse (60 ms total duration). All spectra were transformed and weighted with a 90° shifted sine-bell squared function to 4K x 4K real data points. NOESY and TOCSY spectra were analyzed for solutions with *R* = [drug]/[DNA] = 2.0 and 3.0 for BHM21 (**1**) and BA-41 (**2**), respectively. At these ratios, it was easier to identify the ligand signals, which is important to assign the intermolecular NOE interactions. Proton resonance assignments of the free c-kit21T12T21 sequence were performed on the basis of previous assignments [32]. The chemical shift values of the complexes of **2** and **1** with c-kit21T12T21 are reported in Tables S3 and S4. The assignment of the resonances of **2** in the complex is reported in Table S6. Some aromatic protons of **2** and **1** lie in a crowded region of the oligonucleotide signals and thus could not be assigned.

### 3.5. Molecular Modeling Studies

The two ligands that were the object of this study were optimized as previously described [30], while the coordinates of the c-kit2 promoter form-I were obtained from the NMR structure deposited in the Protein Data Bank (accession code: 2KYP) [32]. The CUDA^®^ version of the GROMACS package [46] with the 53A6 GROMOS force field [47] was used to perform energy minimizations and molecular modeling calculations, while molecular docking experiments were conducted using the Autodock 4.0 software [48]. The molecular docking experiments were performed using the Lamarckian Genetic Algorithm [49], and the Autodock Tool Kit (ADT) [50] was used to further process both the ligands and the c-KIT model. In ADT, the Gasteiger–Marsili charges [51] were loaded on the ligands, while the phosphorus atoms in the DNA were parameterized using the Cornell parameters. The systems were completed by adding the solvation parameters by means of the Addsol utility of Autodock. For each docking run, the initial population consisted of 100 randomly placed individuals, with a maximum number of 250 energy evaluations and a mutation rate of 0.02, a crossover rate of 0.80, and an elitism value of 1. For the local search, 250 independent docking runs were carried out for each ligand by applying the so-called pseudo-Solis and Wets algorithm with a maximum of 250 iterations per local search. The system in the actual docking process was represented by grid maps calculated with Autogrid, with grid dimensions of 80 × 80 × 80 Å, with a spacing of 0.01 Å. The docking results were scored by using the simpler intermolecular energy function based on the Weiner force field, as implemented in Autodock. Results differing by less than 1.0 Å in positional root-mean-square deviation (rmsd) were clustered together and were represented by the result with the most favorable free energy of binding. The complexes obtained in the docking phase were balanced through 5.0 ns of molecular dynamics using the CUDA® version of the GROMACS package running on a dual-Xeon workstation (8 core) equipped with an NVIDIA® GPU containing about 5000 CUDA® cores.

### 3.6. Cell Lines

The human lymphoma cell line SUD4HL1 was obtained from ATCC (CRL-2955) (Rockville, MD, USA) and was grown in RPMI 1640 (Lonza, Verviers, Belgium) supplemented with 10% FBS (Life Technologies, NY, USA), 1% penicillin/streptomycin (Lonza).

### 3.7. Drug Treatment and Cell Growth Inhibition

To evaluate the effects of **2** on the human lymphoma cell line SU-D4HL-1, the IC_50_ was determined. Briefly, 2 × 10^4^ SUD4HL1 cells/well were seeded in 12-well plates in triplicate, and treated the following day. Viable cells were counted using the Trypan blue exclusion method at 24, 48 and 72 h after treatment. As a vehicle, 0.1% DMSO (Mylan, Illinois, USA) was administered. 

### 3.8. Western Blot

Cells were lysed in RIPA buffer, and lysates were separated by SDS-PAGE using precast 4–15% gradient gels (Bio-Rad, California, USA) and transferred to a nitrocellulose membrane (Amersham Protran, GE Healthcare, Germany). Membranes were probed with the following primary antibodies: c-KIT (E-3, SantaCruz Biotechnology, Texas, USA), β-actin (4967, Cell Signaling Technology, Leiden, The Netherlands). Subsequently, protein detection was performed with HRP-conjugated secondary antibodies (Thermo Fisher, Waltham, Massachusetts, USA) and the ECL system (GE Healthcare, Fisher Scientific Italia, Rodano, Italy).

## 4. Conclusions

Recent studies have reported that compounds **1** and **2** interact with the Pu22T14T23 and Pu19A2A11 sequences of the c-MYC promoter [30]. To extend previous results in order to ascertain the detailed mechanism of action of these RNA polymerase I inhibitors, we investigated the ability of compounds **1** and **2** to modulate the conformational equilibria of c-*KIT* G-rich sequences. The NMR and modeling results with c-kit21T12T21 provided evidence that these ligands are stably positioned over the tetrad I at the 3′-end, whereas no interaction appears at the 5′-end. The Kb obtained by fluorescence experiments was similar for both complexes (10^6^ M^−1^). 

Moreover, comparison with the complexes previously obtained with the Pu22T14T23 sequence revealed some differences with the above sequence: two molecules of ligand were located at both the external tetrads, whereas only one molecule can bind the G-quadruplex of c-kit21T12T21. This is in part due to the presence of a “non-canonical” C1:A13 pair, which stacks over guanines 14 and 18 of tetrad III.

Compound **2** showed significant antiproliferative activity in a human lymphoma model, SU-DHL4, known to express substantial levels of c-KIT. However, the partial inhibition of c-KIT expression documented by Western blot analysis suggested that the interaction of compound **2** with the c-KIT promoter is not the primary event associated with its biological activity.

These results contribute to the understanding of the pharmacology of these agents, which apparently exert their antiproliferative activity through a multifaceted mechanism of action.

## Figures and Tables

**Table 1:** Thermodynamic parameters for the folding calculated from the ellipticity vs. temperature traces, considering a two-state process. The uncertainty in T_m_ values was ± 0.7 °C. Uncertainties in ΔH^0^ and ΔS^0^ values were lower than ± 5%. Uncertainties in ΔG^0^ values were lower than ± 10%.

	T_m_ (^o^C)	ΔH (kcal·mol^−1^)	ΔS (cal·K^−1^·mol^−1^)	ΔG_37_ (kcal·mol^−1^)
c-kit21T12T21	58.1	−32	−97	−2.0
c-kit21T12T21 + (**1**)	66.3 (+8.2)	−40	−119	−3.5
c-kit21T12T21 + (**2**)	73.0 (+14.9)	−52	−150	−5.4
Pu22T14T23	77.4	−48	−138	−5.6
Pu22T14T23 + (**1**)	80.6 (+3.2)	−57	−160	−7.0
Pu22T14T23 + (**2**)	80.6 (+3.2)	−49	−139	−6.1

**Table 2:** Intermolecular NOE in the complexes of **2** with c-kit21T12T21. ^a^

3′ Binding Site	
**NOE**	d (Å)^b^
BA-41 (**2**) c-kit21T12T21	(**2**)/c-kit21T12T21
H1 G8H1	4.8
H1 G16H1	4.4
H2 G16H1	5.8
H3 G8H1	4.0
H4 G8H1	4.3
H7 G4H1	5.4
H8 G20H1	5.2
H9 G4H1	6.2
H9 G20H1	4.6

^a^ Measured at 25 °C. Solvent H_2_O-D_2_O (90:10 *v/v*), 5 mM K-phosphate buffer, 20 mM KCl, pH 6.9, *R* = 3.0. ^b^ Distances obtained by molecular modeling.

## Supplementary Materials

The following are available online at https://www.mdpi.com/1422-0067/20/19/4927/s1, Table S1: Binding constants (Kb) and stoichiometries calculated from the fluorimetric titration curves.; Table S2: Inter-residue NOE interactions of c-kit21T12T21 in the complex with BA-41 (**2**) and BMH-21 (**1**).; Table S3: ^1^ H chemical shift values for the complex of BA-41 (**2**) with c-kit21T12T21.; Table S4: ^1^ H chemical shift values for the complex of BMH-21 (**1**) with c-kit21T12T21.; Table S5: Guanine residues involved in the π–π interactions with the polycyclic system of compounds BMH-21 (**1)** and BA-41 (**2**).; Table S6: Chemical shift values of BA-41 in the complex with c-kit21T12T21.; Figure S1: SEC profile recorded for c-kit21T12T21 and Pu22T14T23.; Figure S2: CD spectra recorded along the titration of Pu22T14T23 with BMH-21 (**1**) and BA-41 (**2**).
