# Supplementary material for: Stabilization of c-KIT G-Quadruplex DNA Structures by the RNA Polymerase I Inhibitors BMH-21 and BA-41
Source: Int J Mol Sci. 2019 Oct 4;20(19):4927. doi: 10.3390/ijms20194927 (PMC6801708; doi:10.3390/ijms20194927)
Supplement: Supplementary file 1 [file ijms-20-04927-s001.pdf]

Supplementary Materials

Stabilization of c-KIT G-Quadruplex DNA Structures by the RNA polymerase I inhibitors BMH-21 and BA-41

Stefania Mazzini*, Raimundo Gargallo, Loana Musso, Francesca De Santis, Anna Aviñó, Leonardo Scaglioni, Ramon Eritja, Massimo Di Nicola, Franco Zunino, Annabella Amatulli, Sabrina Dallavalle

Table S1: Binding constants (Kb) and stoichiometries calculated from the fluorimetric titration curves.

Table S2: Inter-residue NOE interactions of c-kit21T12T21 in the complex with BA-41 (**2**) and BMH-21 (**1**).

Table S3: ^{1}H chemical shift values for the complex of BA-41 (**2**) c-kit21T12T21.

Graph S1: Chemical shift difference of DNA protons between the complex of c-kit21T12T21 with (2) R=3.0 and the free c-kit21T12T21: (a) H1/H2/H5/CH3, (b) H6/H8

Table S4: ^{1}H chemical shift values for the complex of BMH-21 (**1**) with c-kit21T12T21.

Graph S2: Chemical shift difference of DNA protons between the complex of c-kit21T12T21 with (1) R=2.0 and the free c-kit21T12T21: (a) H1/H2/H5/CH3, (b) H6/H8

Table S5: Guanine residues involved in the pi-pi interactions with the polycyclic system of compounds BMH-21 (**1**) and BA-41 (**2**).

Table S6: Chemical shift values of BA-41 in the complex with c-kit21T12T21.

Figure S1: SEC profile recorded for Pu22T14T23.

Figure S2: CD spectra recorded along the titration of Pu22T14T23 with BMH-21 (**1**) and BA-41 (**2**).

Table S1. Binding constants (Kb) and stoichiometries calculated from the fluorimetric titration curves in Figures S3 and S4

Ligand	Overall ligand:DNA stoichiometry	Kb (M^{-1})	
BA-41 (**2**)	3:1	$10^{6.4\pm0.4}$ (1 binding site)	$10^{6.2\pm0.4}$ (2 binding sites)
BMH-21 (**1**)	2:1	$10^{6.4\pm0.44}$ (1 binding site)	$10^{5.9\pm0.24}$ (1 binding site)

Table S2. Inter-residue NOE interactions of c-kit21T12T21 in the complex with BA-41 (**2**) and BMH-21 (**1**).

G-tetrad I	*G-tetrad II*	*Tetrad III*
G4H1…..G8H8	G3H1….G7H8	G18H1….G2H8
G8H1…G16H8	G7H1...G15H8	G14H1….G18H8
G16H1…G20H8	G15H1…G19H8	G6H1….G14H8
G20H1…G4H8	G19H1…G3H8	G2H1…..G6H8

Table S3. ^{1}H chemical shift values for the complex of BA-41 (**2**) with c-kit21T12T21.[a]

complex of **BA-41**/c-kit21T12T21[b]						
c-kit	H1/H2/H5/CH_3	$\Delta\delta^c$	H6/H8	$\Delta\delta^c$	H1'	H2'-H2''
C1	6.10	+0.55	7.59	+0.09		
G2	11.15	-0.81	7.99	-0.21	6.01	
G3	10.74	-0.56	7.63	-0.05	6.03	
G4	10.32	-0.83	7.63	-0.12		
C5	6.20	0.00	8.00	-0.04	6.48	2.40, 2.70
G6	10.83	-0.69	7.77	-0.25	5.94	
G7	10.93	-0.65	7.70	-0.32	5.93	
G8	10.44	-0.87	7.71	-0.08		
C9	5.95	-0.15	7.85	+0.23	6.00	
G10	n.d.	-	n.d.			
C11	6.10	+0.04	7.95	+0.05	6.32	
T12	1.60	-0.30	7.45	-0.15	5.99	1.90, 2.13
A13	n.d.	-	8.26	+0.16	6.24	2.70,2.90
G14	11.07	-0.87	7.82	-0.44	6.01	
G15	10.79	-0.59	7.63	-0.24		
G16	10.34	-0.70	7.68	-0.12	6.33	
A17	n.d.	-	8.55	+0.04	6.62	2.92, 2.92
G18	10.97	-0.85	7.83	-0.26		
G19	10.96	-0.60	7.72	-0.37		
G20	10.47	-0.78	7.65	-0.05	6.24	
T21	1.70	+0.20	7.51	+0.36	6.33	2.30, 2.30

[a] Measured at 25°C in ppm (δ) from external DSS. Solvent H_2O-D_2O (90:10 v/v), 5 mM phosphate buffer, 20 mM KCl, pH 6.9. [b]R = 3.0. [c]$\Delta\delta = \delta_{bound} - \delta_{free}$

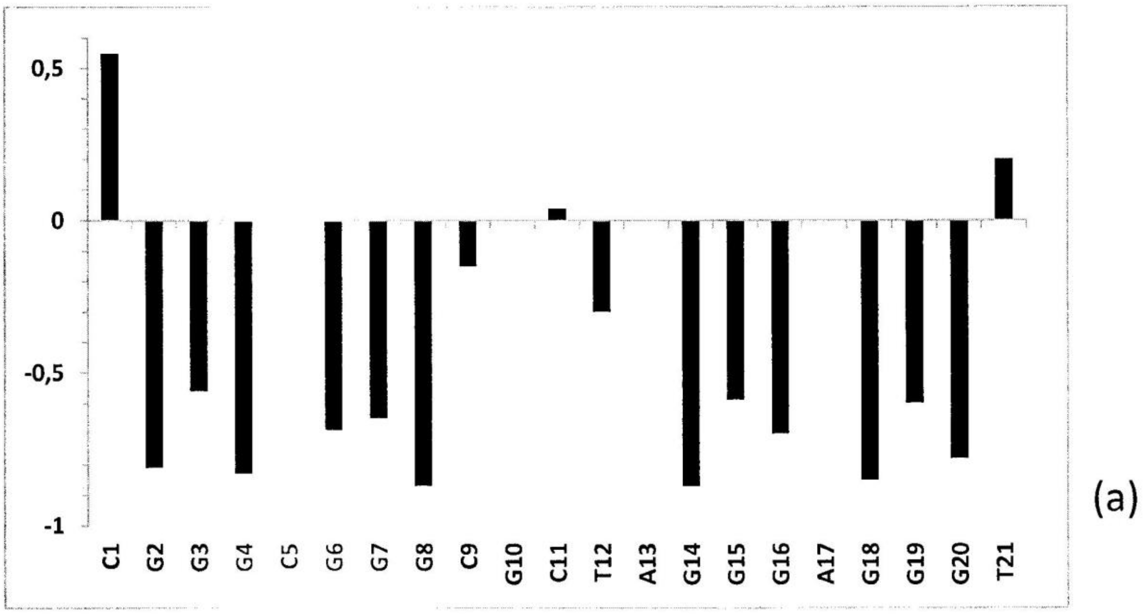

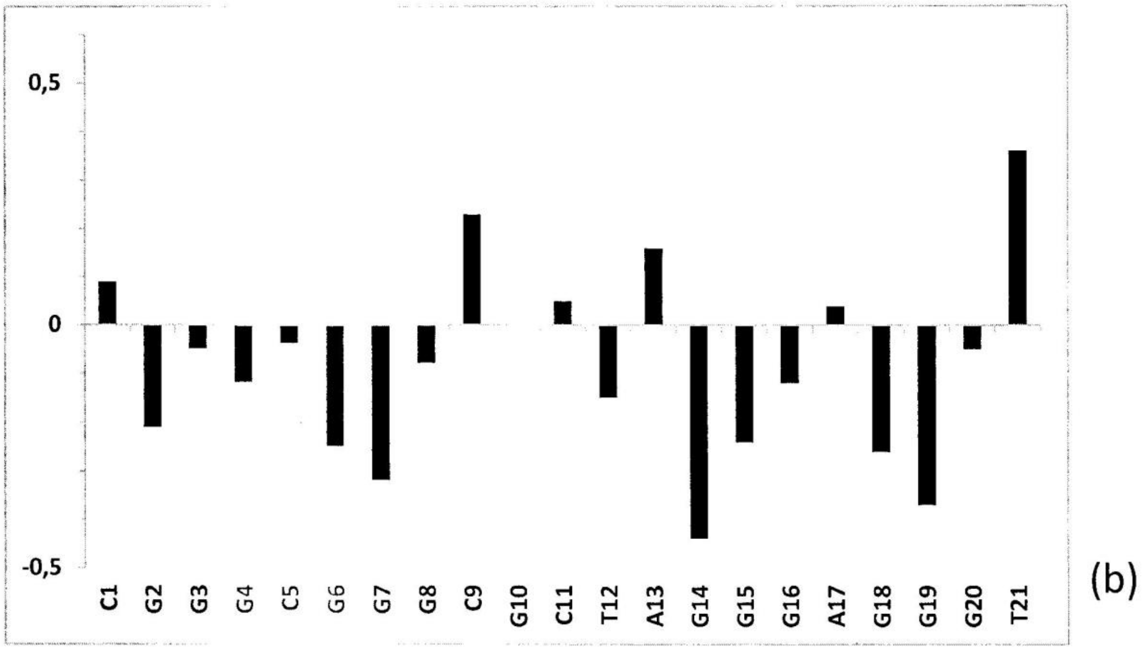

Graph S1: Chemical shift difference of DNA protons between the complex of c-kit21T12T21 with **(2)** R=3.0 and the free c-kit21T12T21: (a) H1/H2/H5/CH_3, (b) H6/H8

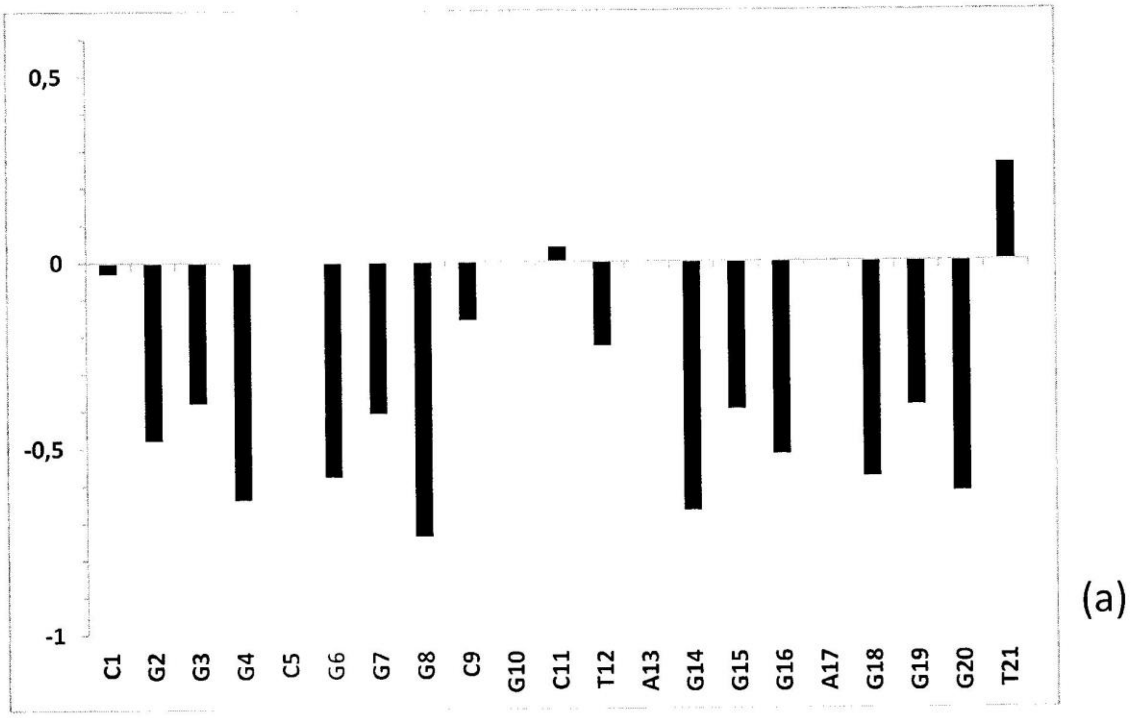

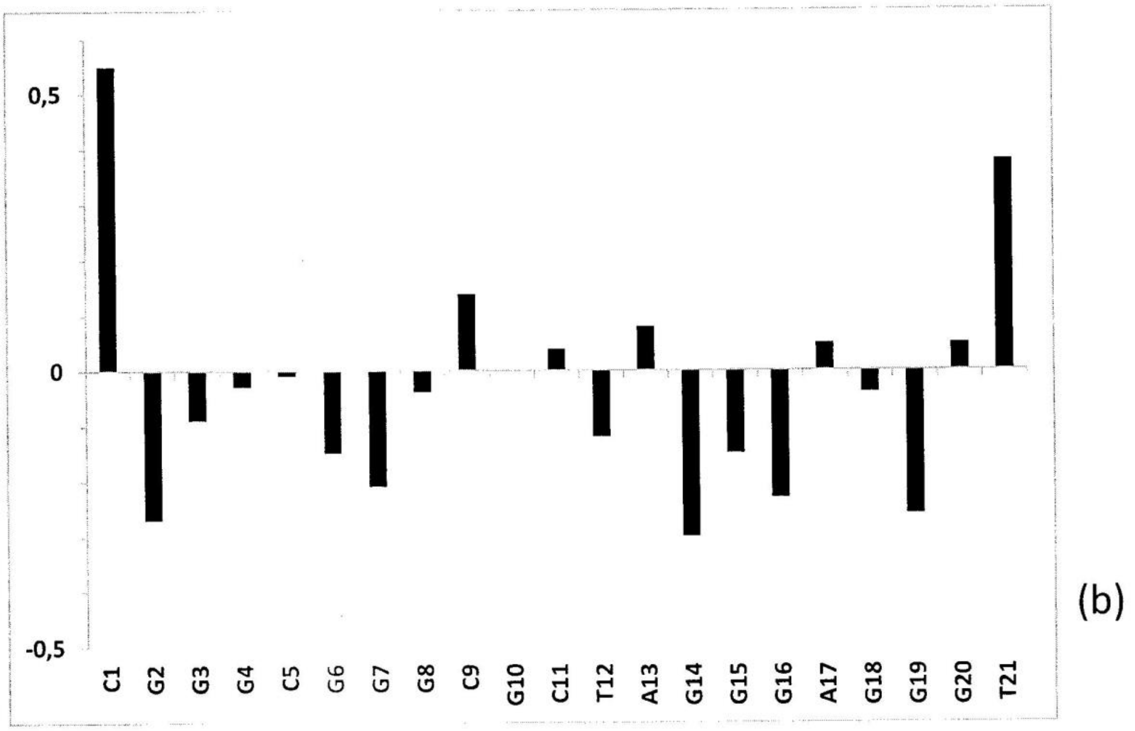

Graph S2: Chemical shift difference of DNA protons between the complex of c-kit21T12T21 with **(1)** R=2.0 and the free c-kit21T12T21: (a) H1/H2/H5/CH_3, (b) H6/H8

Table S4. ^1H chemical shift values for the complex of BMH-21 (**1**) with c-kit21T12T21.[a]

complex of **BMH21**/c-kit21T12T21[b]				
c-kit	H1/H2/H5/CH_3	Δδ[c]	H6/H8	Δδ[c]
C1	6.10	-0.03	7.53	+0.55
G2	11.44	-0.48	7.93	-0.27
G3	10.92	-0.38	7.69	-0.09
G4	10.51	-0.64	7.72	-0.03
C5	6.20	0.00	8.03	-0.01
G6	11.06	-0.58	7.87	-0.15
G7	11.17	-0.41	7.81	-0.21
G8	10.57	-0.74	7.75	-0.04
C9	5.94	-0.16	7.76	+0.14
G10	n.d.	-	n.d.	-
C11	6.10	+0.04	7.94	+0.04
T12	1.67	-0.23	7.48	-0.12
A13	n.d.	-	8.25	+0.08
G14	11.27	-0.67	7.96	-0.30
G15	10.98	-0.40	7.72	-0.15
G16	10.52	-0.52	7.75	-0.23
A17	n.d.	-	8.56	+0.05
G18	11.40	-0.58	8.05	-0.04
G19	11.17	-0.39	7.83	-0.26
G20	10.63	-0.62	7.75	+0.05
T21	1.76	+0.26	7.53	+0.38

[a] Measured at 25°C in ppm (δ) from external DSS. Solvent H_2O-D_2O (90:10 v/v), 5 mM phosphate buffer, 20 mM KCl, pH 6.9. [b] R=2.0. [c] $\Delta\delta = \delta_{bound} - \delta_{free}$

Table S5. Guanine residues involved in the pi-pi interactions with the polycyclic system of compounds BMH-21 (**1**) and BA-41 (**2**)

rings	A	B	C	D
BMH-21 (**1**)	G16	G8, G16	G4, G8	G4
BA-41 (**2**)	G8, G16	G8, G16	G4	G4

Table S6. Chemical shift values of BA-41 in the complex with c-kit21T12T21.[a]

complex of BA-41/c-kit21T12T21	
H1	7.10
H2	6.83
H3	6.94
H4	7.16
H7	7.98
H8	6.70
H9	8.22

[a] Measured at 25°C in ppm (δ) from external DSS. Solvent H_2O-D_2O (90:10 v/v), 5 mM K-phosphate buffer, 20 mM KCl, pH 6.9, R = 3.0.

	MW	log(MW)	tR exp.	tR calc.	Variat. (%)
c-kit21T12T21	6609.3	3.82	10.41	10.92	-4.64%
	6609.3	3.82	11.02	10.92	0.95%
Pu22T14T23	7041.6	3.85	10.76	10.86	-0.92%
	7041.6	3.85	11.61	10.86	6.90%

Retention times and molecular weights of the c-kit21T12T21 and Pu22T14T23 according to the calibration with Tx standards.

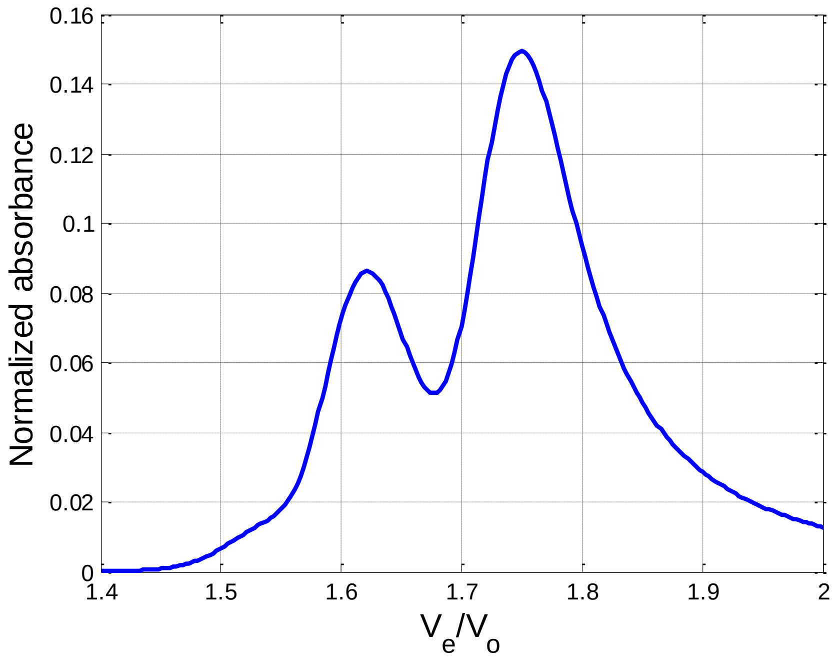

Figure S1. SEC profile recorded for Pu22T14T23. The peaks at V_e/V_0 ratios equal to 1.62 and 1.75 were related to linear and monomeric structure, respectively, according to reference [32]. The void volume was 5.30 mL.

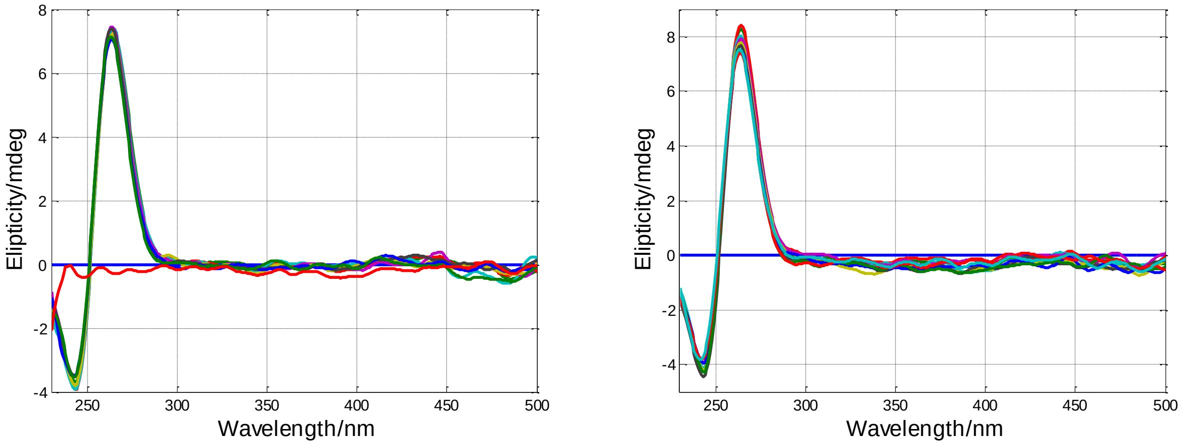

Figure S2. CD spectra recorded along the titration of Pu22T14T23 with (a) BMH-21 (**1**) and (b) BA-41 (**2**). In both cases, the medium consisted of 20 mM phosphate buffer (pH 7.0) and 5 mM KCl. DNA concentration was 1 μM. Ligand concentration ranged from 0 to 3 μM. Experiments were carried out at 25 °C.
